# A database of human gait performance on irregular and uneven surfaces collected by wearable sensors

**DOI:** 10.1038/s41597-020-0563-y

**Published:** 2020-07-08

**Authors:** Yue Luo, Sarah M. Coppola, Philippe C. Dixon, Song Li, Jack T. Dennerlein, Boyi Hu

**Affiliations:** 1 Department of Industrial and Systems Engineering, University of Florida, Gainesville, United States; 2 John Hopkins University School of Medicine, Baltimore, United States; 3 School of Kinesiology and Physical Activity Sciences, Faculty of Medicine, University of Montreal, Montreal, Canada; 4 Research Center of the Sainte-Justine University Hospital, Montreal, Canada; 5 Bouvé College of Health Sciences, Northeastern University, Boston, United States; 6 Department of Environmental Health, Harvard T.H. Chan School of Public Health, Boston, United States

**Keywords:** Public health, Risk factors

## Abstract

Gait analysis has traditionally relied on laborious and lab-based methods. Data from wearable sensors, such as Inertial Measurement Units (IMU), can be analyzed with machine learning to perform gait analysis in real-world environments. This database provides data from thirty participants (fifteen males and fifteen females, 23.5 ± 4.2 years, 169.3 ± 21.5 cm, 70.9 ± 13.9 kg) who wore six IMUs while walking on nine outdoor surfaces with self-selected speed (16.4 ± 4.2 seconds per trial). This is the first publicly available database focused on capturing gait patterns of typical real-world environments, such as grade (up-, down-, and cross-slopes), regularity (paved, uneven stone, grass), and stair negotiation (up and down). As such, the database contains data with only subtle differences between conditions, allowing for the development of robust analysis techniques capable of detecting small, but significant changes in gait mechanics. With analysis code provided, we anticipate that this database will provide a foundation for research that explores machine learning applications for mobile sensing and real-time recognition of subtle gait adaptations.

## Abstract (summary)

**Table:** 

Measurement(s)	Gait
Technology Type(s)	Sensor Device
Factor Type(s)	surface • age • sex • height • body mass
Sample Characteristic - Organism	Homo sapiens

Machine-accessible metadata file describing the reported data: 10.6084/m9.figshare.12505022

## Background & Summary

Gait analysis is the science of functional assessment of human locomotion, and it has been applied in multiple areas such as medicine, sport, and ergonomics with promising results^1–3^. One specific successful application of gait analysis is to assess fall risk exposure and prevent falling injuries^4^. Fall risk is associated with multiple factors including human characteristics, health conditions, and the physical environment^5^. In particular, irregular walking surfaces in the outdoor built and natural environment expose people to potential fall injuries^6^. Unfortunately, traditional gait analysis requires expensive engineering technologies that are time and labor intensive, especially when the analysis involves heuristic hand-crafted feature extraction^7–9^. To overcome this limitation, machine learning methods are increasingly being integrated into gait and posture related investigations^10–12^.

This data descriptor aims to contribute to machine learning research of gait performance when walking in different outdoor environments, which has surprisingly been limited in previous literature. Previous work has shown that gait adaptations utilized when walking on irregular surfaces may reflect reduced stability and increased fall risk^13–15^. However, one limitation of such previous studies is that they were conducted in simulated laboratory environments and thus lack real world validity. With the recent development of wearable motion tracking technologies such as Inertial Measurement Units (IMU), we now have the capability to extend gait analysis into outdoor settings to maximize ecological validity.

In order to develop accurate, robust and generalizable machine learning algorithms to recognize subtle gait alterations, it is necessary to have sufficient amounts of properly annotated data. Unfortunately, very limited gait related data sets are publicly accessible. Among these, most were primarily generated for human activity recognition purposes so the activity tasks included have a very broad spectrum of coverage^16–24^. For example, gait is usually one category accompanied by other activities that have substantial differences (sitting, lying down, climbing stairs, running, etc.). Subtle gait alterations due to internal/external factors have never been considered or properly annotated in existing public data sets. A second category of data sets are focused on utilizing human gait performance as a biometrics characteristic for human identification^12,25–30^. Therefore, creators of those data sets usually only considered between subject differences and only collected short duration of gait trials from each participant which is not sufficient to train advanced machine learning models. Furthermore, the environmental conditions in which these data were collected are not always reported in sufficient detail. In order to advance machine learning for the recognition of human gait changes caused by walking surface characteristics, there is an urgent need to create large data sets that have an exhaustive set of walking surfaces representative of the real environment outside the laboratory, preferably with wearable and non-intrusive sensors.

Therefore, in this descriptor, we present a publicly accessible data set collected with wearable motion sensors where participants walked on different real-world outdoor surfaces. We anticipate that this data set will provide a foundation for subsequent research that explores the application of machine learning to mobile sensing and real-time recognition of subtle gait adaptations.

## Methods

### Participants

Thirty young participants with no reported neurological or musculoskeletal conditions that affected their gait or posture and no history of falling injuries in the previous two years volunteered for this study. The sample of participants is in proximity to normal urban US campus. Their anthropometry information is provided in Table 1. The Harvard and Northeastern Institutional Review Boards approved this study and all participants provided written consent.

**Table 1:** Anthropometry information of participants.

Participant	Age	Sex	Height (cm)	Body mass (kg)
1	28	F	154.5	49.1
2	24	F	158.6	54.1
3	22	F	167	53.6
4	22	F	166	56
5	23	F	168.2	61.4
6	33	M	175	99
7	27	M	184	75.3
8	18	M	187	82.3
9	22	F	162.1	53.6
10	19	F	162	61.7
11	28	M	180	70.4
12	18	M	177.9	81
13	22	F	174.2	58.6
14	19	F	66.5	67.3
15	19	M	181	72.4
16	31	M	176	101.2
17	19	F	173	73.9
18	30	M	165.4	82.9
19	32	F	165	53
20	22	F	167.1	74.8
21	19	M	169	73.9
22	22	M	178.5	80.2
23	24	F	179.6	61.6
24	26	F	174.6	62.5
25	22	F	157	61.6
26	22	M	175.6	66
27	22	M	192.7	85.9
28	22	M	180	91.1
29	26	M	172	78.1
30	22	M	188	84.4
Summary	23.5 (4.2)	15M, 15F	169.3 (21.5)	70.9 (13.9)

### Data collection

**Fig. 1:**
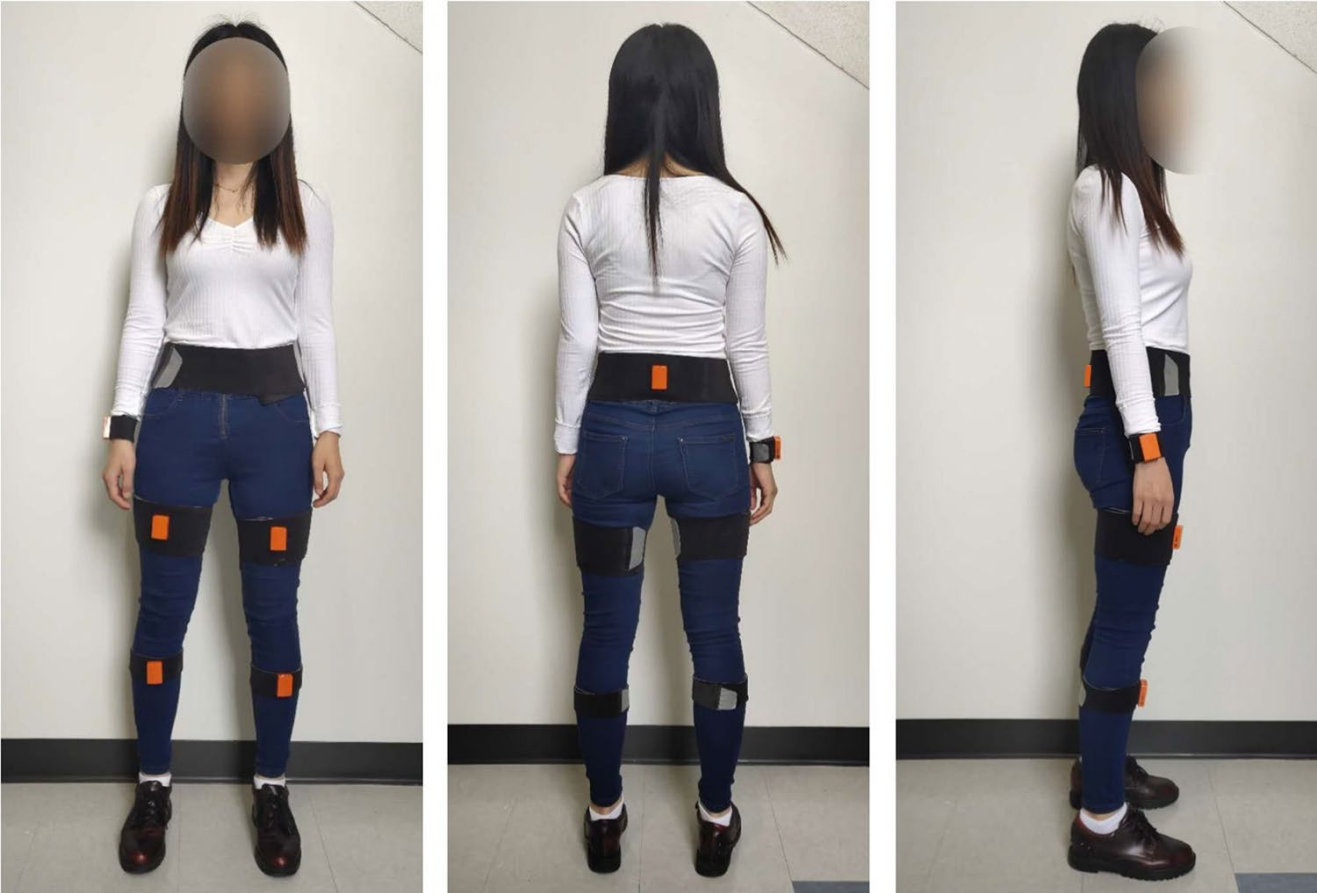
Sensor placement setup.

Participants performed several walking trials over nine different surfaces while wearing six IMU sensors (MTw Awinda, Xsens, Enschede, Netherlands). The sensors were secured to the body using the bands provided by the manufacturer such that they were: 1) centered on the wrist on the dorsal forearm, 2 & 3) centered on both the anterior thighs, 4 & 5) centered 5 cm above the bony processes of both ankles, and 6) posterior level of L5/S1 joint (Fig. 1).

**Fig. 2:**
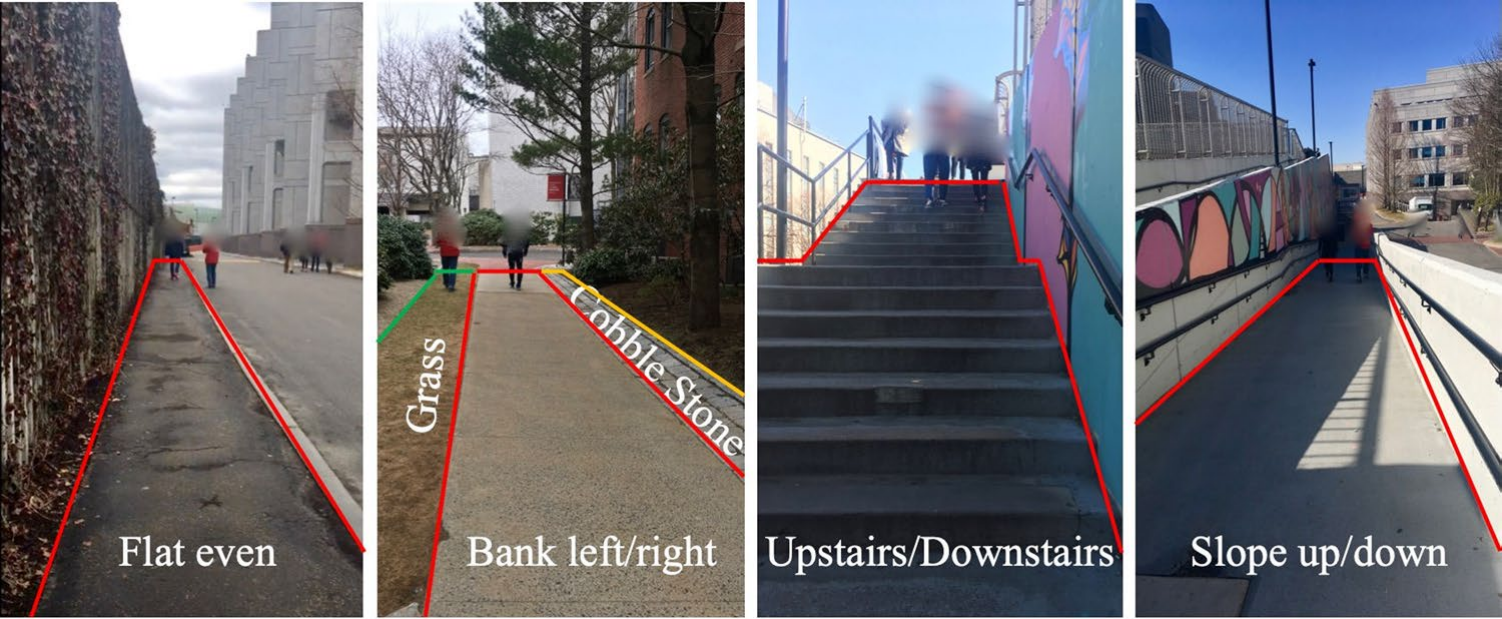
Measurement sites for walking trials.

Researchers palpated participant’s bones to place the sensors. Participants were instructed to face southwest and perform a sensor calibration procedure three times prior to the experimental trial collection. The calibration procedure was: 1) line up directly centered with experiment computer; 2) forward trunk flexion about 30 degrees 3 times; 3) raise right arm 3 times; 4) raise right leg three times; 5) raise left leg three times. A researcher performed these movements with the participant. The calibration data are also included in this data set. The nine walking surfaces were: 1) flat even (horizontal, 0 grade, paved); 2) up stairs (cement); 3) down stairs (cement); 4) sloped up (cement); and 5) sloped down (cement) 6) grass; 7) banked left (paved); 8) banked right (paved); 9) uneven stone brick (Fig. 2).

**Fig. 3:**
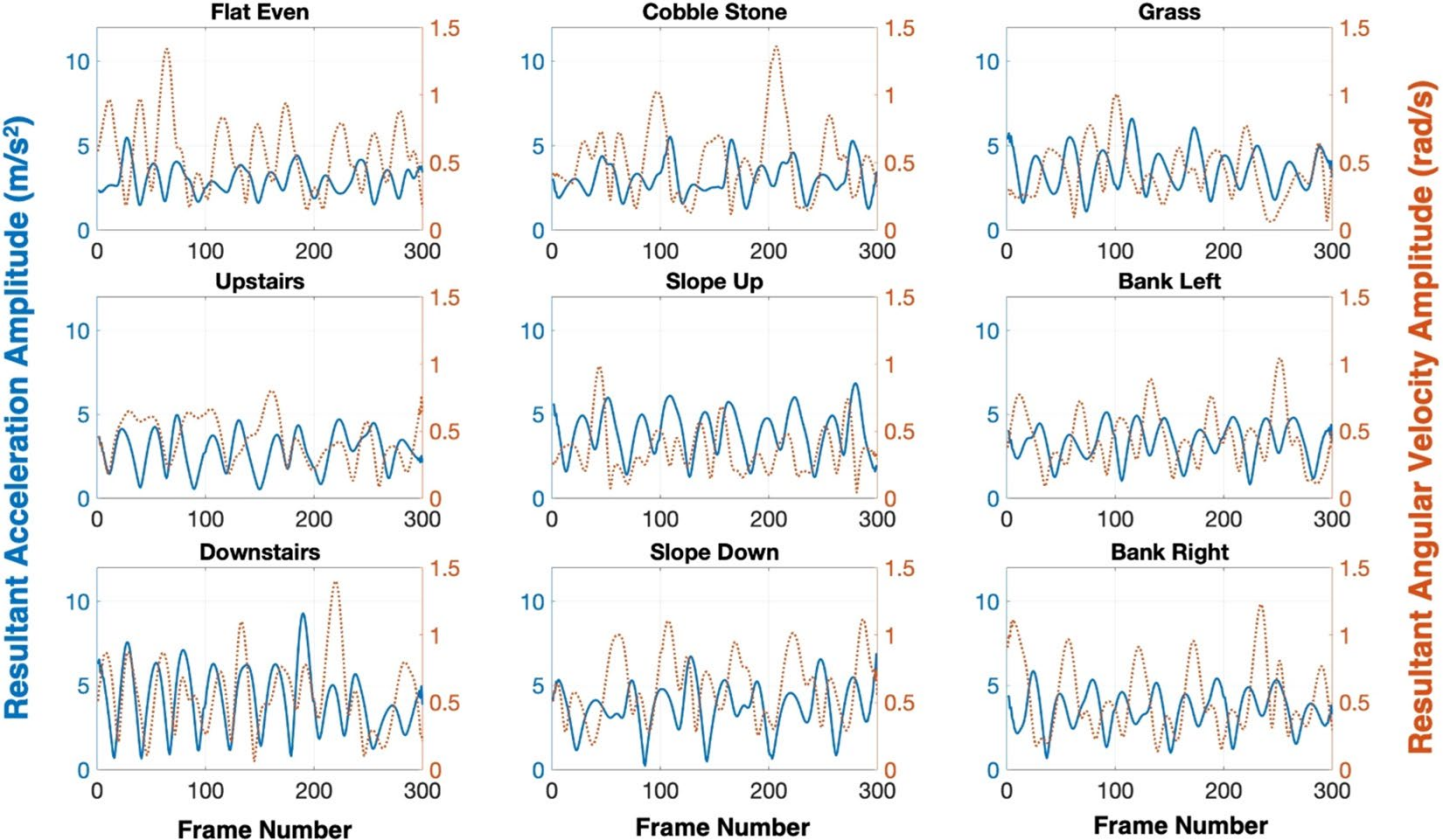
Signal pattern of trunk sensor on different walking surfaces: resultant acceleration amplitude (*m*/*s*^2^, blue solid lines) and resultant angular velocity amplitude (*rad*/*s*, red dotted lines) from subject #1.

Participants were instructed to walk at their normal pace and to let their arms swing naturally. Participants stood still at the starting position and waited for the verbal cue from a researcher to start their walking trials. Each walking trial lasted for 16.4 ± 4.2 seconds until stop. Within each trial, walking was performed by participants without changes of direction (i.e. straight walking). Between trials, only walking on flat even, grass, and uneven stone brick were conducted with direction changes every other trial (i.e. walking forward for the first trial and walking back for the next trial). Surfaces were presented in a randomized order and adequate rest was provided to prevent fatigue between trials. Participants walked six times on each of these surfaces, and a researcher walked next to them with the experimental data capture machine to ensure a strong signal connection. A summary of the data collection conditions includes weather (‘N/A’ was filled if weather was not recorded), temperature, and time of day for each participant is provided in Table 2.

**Table 2:** Data collection conditions.

Participant	Temperature (°C)	Wind (*m*/*s*)	Weather	Time of day
1	−1.1	11.2	N/A	Morning (9:30 am)
2	4.4	8.9	Sunny	Afternoon (2:30 pm)
3	4.4	8.0	Cloudy	Noon
4	0	7.6	Sunny	Morning (9:30 am)
5	5.0	5.8	Sunny	Afternoon (2 pm)
6	6.7	3.1	Sunny	Afternoon (6 pm)
7	2.8	2.2	Cloudy	Morning (8 am)
8	2.2	3.1	N/A	Morning (11 am)
9	11.7	6.3	Partly cloudy	Afternoon (2:40 pm)
10	16.7	4.0	Partly cloudy	Morning (10 am)
11	6.1	8.5	Sunny	Morning (9 am)
12	7.2	8.0	Partly cloudy	Morning (10:30 am)
13	9.4	8.0	Cloudy	Afternoon (3:30 pm)
14	7.8	7.6	Cloudy	Afternoon (noon)
15	10.6	7.6	N/A	Afternoon (4 pm)
16	10.0	6.7	N/A	Afternoon (6 pm)
17	8.9	8.0	N/A	Afternoon (1 pm)
18	8.3	5.8	Sunny	Morning (10 am)
19	10.0	5.8	Sunny	Morning (11 am)
20	12.2	4.5	Sunny	Morning (11:30 am)
21	12.8	5.4	Sunny	Afternoon (1 pm)
22	14.4	4.5	Cloudy	Morning (9:30 am)
23	15.0	3.1	Cloudy	Morning (11:30 am)
24	20.0	4.5	Sunny	Afternoon (2 pm)
25	22.8	4.9	Sunny	Afternoon (5:30 pm)
26	20.0	6.7	Partly cloudy	Morning (10:30 am)
27	9.4	0.4	Cloudy	Morning (9:30 am)
28	17.8	3.1	Cloudy	Afternoon (4 pm)
29	10.6	4.0	Partly cloudy	Afternoon (5 pm)
30	15.6	2.2	Cloudy	Morning (9:40 am)

### Data processing

Wearable data were collected using the MTw Awinda software (Xsens, Enschede, Netherlands). The sampling frequency was set at 100 Hz. Raw sensors’ outputs were synchronized by the software and then exported to a standard txt file format. Subsequently, all the data files were imported and processed under MATLAB (R2019a, The MathWorks, Natick, USA). Trajectories were smoothed using a 2^nd^ order Butterworth low pass filter with a 6 Hz cut-off frequency. Figure 3 is presented to give an example of the filtered signal pattern of the trunk sensor while walking on different surfaces.

## Data Records

### Raw data

All raw data files exported from MTw are stored as .txt format and have been uploaded into figshare^31^ to provide free accessibility to the public. A total of 10,260 (30 participants * 57 trials * 6 sensors) files are available from the database. Files are grouped by folders with labels from 1–30 representing the participant number (30 participants in total). Each file was named systematically as ‘#-000_00B432**.txt’, where ‘#’ represents the walking surface condition (Table 3) and ‘**’ represents the sensor location (Table 4). For example, file ‘9-000_00B432CC.txt’ stands for the trunk sensor (‘CC’) data while walking on the flat even surface (‘9’) for all participants. Furthermore, for each trial there was a .mtb file (i.e. binary motion tracker file).

**Table 3:** Table for walking surface condition and sample duration (across all participants).

Trial number (#)	Walking surface condition	Sample duration (s) Mean (standard deviation)
1–3	Calibration (CALIB)	19.29 (3.14)
4–9	Flat even (FE)	13.55 (2.19)
10–15	Cobble stone (CS)	16.12 (1.93)
16,18,20,22,24,26	Upstairs (StrU)	12.48 (1.17)
17,19,21,23,25,27	Downstairs (StrD)	11.84 (1.42)
28,30,32,34,36,38	Slope up (SlpU)	22.70 (1.89)
29,31,33,35,37,39	Slope down (SlpD)	22.77 (2.22)
40,42,44,46,48,50	Bank left (BnkL)	16.06 (1.90)
41,43,45,47,49,51	Bank right (BnkR)	16.29 (1.67)
52–57	Grass (GR)	14.48 (1.52)

**Table 4:** Table for sensor locations of each trial based on last 2 digits of filenames.

Orange Sensor number/**	Sensor location
CC.txt	Trunk
95.txt	Wrist
93.txt	Right thigh
8B.txt	Left thigh
9B.txt	Right shank
B6.txt	Left shank

Sensors’ outputs (e.g. 3D acceleration, 3D gyroscope data) as well as the recording information (e.g. start time, update rate, filter profile, and firmware version) are stored in each file with labels. The average duration for each surface condition (across all participants) is summarized in Table 3. A comprehensive description of the data structure and variable labels are given in Table 5.

**Table 5:** Data stored in .txt files (all variables are with dimension n x 1).

Labels	Unit	Description
PacketCounter	*N*/*A*	Packet counter, value will be same if data frames were recorded at the same time (increase 1 unit per data frame)
SampleTimeFine	*N*/*A*	Not recorded in this study
Acc_X	*m*/*s*^2^	Acceleration in the vertical direction (w/gravity)
Acc_Y	*m*/*s*^2^	Acceleration in the medio-lateral direction (w/gravity)
Acc_Z	*m*/*s*^2^	Acceleration in the anterior-posterior direction (w/gravity)
FreeAcc_X	*m*/*s*^2^	Acceleration in the vertical direction (w/o gravity)
FreeAcc_Y	*m*/*s*^2^	Acceleration in the medio-lateral direction (w/o gravity)
FreeAcc_Z	*m*/*s*^2^	Acceleration in the anterior-posterior direction (w/o gravity)
Gyr_X	*rad*/*s*	Rate of turn along the vertical direction
Gyr_Y	*rad*/*s*	Rate of turn along the medio-lateral direction
Gyr_Z	*rad*/*s*	Rate of turn along the anterior-posterior direction
Mag_X	*a*.*u*.	3D magnetic field in the vertical direction
Mag_Y	*a*.*u*.	3D magnetic field in the medio-lateral direction
Mag_Z	*a*.*u*.	3D magnetic field in the anterior-posterior direction
VelInc_X	*m*/*s*	Delta_velocity (dv) in the vertical direction
VelInc_Y	*m*/*s*	Delta_velocity (dv) in the medio-lateral direction
VelInc_Z	*m*/*s*	Delta_velocity (dv) in the anterior-posterior direction
OriInc_q0	*N*/*A*	Delta_quaternion (q0)
OriInc_q1	*N*/*A*	Delta_quaternion (q1)
OriInc_q2	*N*/*A*	Delta_quaternion (q2)
OriInc_q3	*N*/*A*	Delta_quaternion (q3)
Roll	*deg*	Euler angles in XYZ Earth fixed type (roll)
Pitch	*deg*	Euler angles in XYZ Earth fixed type (pitch)
Yaw	*deg*	Euler angles in XYZ Earth fixed type (yaw)

### Processed data

A processed data file was also provided as a .mat format (data file format of MATLAB) in the repository. Raw sensor data from 30 participants were aggregated into one single file with participant as the first layer and sensor as the second layer.

The outline of the MATLAB script is described as following: 1. import the raw txt files; 2. apply Butterworth low-pass filter (2nd order, cutoff frequency: 6 Hz, sampling frequency: 100 Hz); 3. count the missing frames; 4. export processed data into .mat file.

## Technical Validation

### Sensor placement

Participants were required to wear tight clothes during the experiment to prevent sensor movement. As described in the procedures (see Data Collection), the wearable sensor placement followed the instructions available in the manufacturer’s documentation. In addition, before each experiment, the signal quality of each IMU sensor was manually verified through the system’s acquisition software. IMU sensors were positioned by the same researchers (Authors BH and SC) for consistency.

### Missing data

The trial-wise data missing rate is recorded in the database for each participant (under the second layer of the .mat file). Due to transmission errors between the data collection computer and the IMU sensors, some data frames/packages were dropped. However, we have confirmed that missing data is not a major issue for this data set, only a small fraction of data packages were dropped (0.23% ± 0.69%). Data missing rate is summarized by sensor location in Table 6 and by walking surface in Table 7.

**Table 6:** Table for data missing rate by sensor locations.

Sensor location	Missing rate Mean (standard deviation)
Trunk	0
Wrist	0.13% (0.13%)
Right thigh	0.19% (0.18%)
Left thigh	0.93% (4.08%)
Right shank	0.08% (0.08%)
Left shank	0.06% (0.05%)

**Table 7:** Table for data missing rate by walking surfaces.

Sensor location	Missing rate Mean (standard deviation)
Calibration (CALIB)	0
Flat even (FE)	0.17% (0.26%)
Cobble stone (CS)	0.36% (1.67%)
Upstairs (StrU)	0.59% (3.04%)
Downstairs (StrD)	0.66% (3.03%)
Slope up (SlpU)	0.02% (0.05%)
Slope down (SlpD)	0.10% (0.20%)
Bank left (BnkL)	0.16% (0.23%)
Bank right (BnkR)	0.30% (0.42%)
Grass (GR)	0.12% (0.17%)

### Comparison with published data sets

The age of the participants differed significantly from previously published data sets, which varied from ages 2 to 78 years^18–20,22,24–27,29^, whereas this data set only included young adults. The number of participants of previous data sets also varied significantly from 8 to 744. Subject number is an important technical component for database selection considering the need for large amounts of data during machine learning model training. Nevertheless, it also obscures the merit of data sets that have relatively few participants, but longer recording lengths. For example, although Ravi *et al*.^23^ only recruited 10 participants in their study, a total of 30 hours of data were collected using different models of smartphones with an unconstrained phone placement setting. The data set can be treated as a suitable data resource of models designed for real-world application in which the models and placement of smartphones are always unspecified. Our data set includes 30 participants and each one has a relatively large amount of data collected. The current data set is well aligned with previous similar data sets. When using these data sets for gait-related machine learning model development, we should be aware that the relative homogeneous samples might restrict the generalizability to more heterogeneous data in terms of age distribution.

The annotation of the ground truth for recorded activities is also important for publicly accessible data sets because it is needed to validate the predicted outcome. Most of the previous similar data sets have documented the types of activities participants performed. Among them, many include walking records on different surfaces (walking on concrete/grass field, walking upstairs/downstairs, etc.)^16,18–22,24,26,27^. Compared to them, the current data set provides a larger amount of irregular walking surfaces. Machine learning algorithm developers could benefit from the diversified walking records contained in the present data set.

Although some parameters about testing sites (e.g. the grade of the slope and the stair dimensions) were not systematically surveyed during the data collection phase, we believe they represent common public architecture features. To further improve the usability of the data, more details about measurement sites will be provided in the GitHub and publicly accessible data description in the future.

## Usage Notes

Previous literature has shown that IMUs are a valid tool for measuring subtle changes in gait kinematics and the performance is as sensitive as the current standard in kinematic tracking (i.e. optical motion capture)^32^. To support a range of users in accessing the data set, other than raw data, processed data are provided in .mat format in the data repository. The .mat data file is readable by both Python and MATLAB environments.

Existing Python and MATLAB open-source tools focused on gait and human motion kinematics could be used to analyze this data set. GaitPy provides python functions to read accelerometry data and estimate the clinical characteristics of gait (https://pypi.org/project/gaitpy/). It could be a complementary tool when utilizing this data set. For MATLAB, the Kinematics and Inverse Dynamics toolbox (https://www.mathworks.com/matlabcentral/fileexchange/58021-3d-kinematics-and-inverse-dynamics) can be utilized in investigating joint kinematics and dynamics. Moreover, biomechZoo, which help users analyze, process, and visualize motion data from various sensors^33^ could support researchers aiming to explore this data set.

## Data Availability

The custom MATLAB script to process data is provided on the following Github repository: https://github.com/UF-ISE-HSE/UnevenWalkingSurface. A Python script (python_version.py) was also provided for converting the processed data into Python compatible format. The .h5py file can be directly use as a standard file object in Python to process.
